# Individual and joint associations of obesity and metabolic health parameters on arterial stiffness: Evidence from the UK Biobank

**DOI:** 10.1111/dom.16090

**Published:** 2024-11-25

**Authors:** Roshan A. Ananda, Bethlehem Solomon, Kausik K. Ray

**Affiliations:** ^1^ School of Public Health Imperial College London London UK; ^2^ Department of General Medicine Box Hill Hospital Melbourne Victoria Australia

**Keywords:** arterial stiffness, metabolic health, obesity, preventive cardiology, UK Biobank, vascular health

## Abstract

**Aims:**

There is conflicting evidence regarding whether excess adiposity without metabolic abnormalities reflects a truly benign phenotype. This study evaluated the independent and joint associations of the presence of excess adiposity and metabolic abnormalities on arterial stiffness.

**Materials and Methods:**

Participants in UK Biobank with body mass index (BMI) and arterial stiffness index (ASI) recorded between 2006 and 2010, free from cardiovascular diseases and not underweight (BMI <18.5 kg/m^2^) were included. The primary outcome was severity of ASI analysed using multivariate‐adjusted linear regression.

**Results:**

Of 162 590 participants, 42.5% were overweight and 24.4% were obese. Within the normal BMI strata, 50.7% had ≥1 metabolic abnormality. Compared to individuals with normal BMI and no metabolic abnormality (reference group), increased BMI or metabolic abnormalities were similarly associated with higher ASI: normal BMI with metabolic abnormalities (adjusted β‐coefficient and 95% CI, 0.35; 0.30–0.40); overweight without metabolic abnormalities (0.32; 0.26–0.37). Individuals with obesity and no metabolic abnormality had higher ASI (0.65; 0.57–0.74) but was lower than individuals with overweight and metabolic abnormalities (0.80; 0.75–0.84). Individuals with obesity and metabolic abnormalities had the highest ASI (1.07; 1.02–1.12) among all six metabolic combinations, *p* < 0.001 for each versus reference group. Sensitivity analysis suggested higher ASI with increasing number of metabolic abnormalities within BMI categories and higher ASI in the presence of abdominal obesity within metabolic categories.

**Conclusions:**

Excess adiposity and metabolic abnormalities are independently associated with increased arterial stiffness to a similar degree, suggesting that metabolically healthy individuals with overweight and obesity are not benign groups. This reinforces the need to prevent excess adiposity and consider primary prevention strategies even before metabolic abnormalities emerge.

## 1 INTRODUCTION

Obesity is an established modifiable risk factor for cardiovascular disease (CVD), and its prevalence has been increasing globally over several decades.^1^, ^2^ Insulin resistance underpins many of the metabolic abnormalities which cluster in individuals with obesity, including hypertension, diabetes, and dyslipidaemia, which are known to independently associate with cardiovascular risk.^3^, ^4^, ^5^ However, these metabolic abnormalities are absent in approximately half of individuals with obesity, and who have been referred to as ‘metabolically healthy obesity’ (MHO).^4^, ^6^, ^7^, ^8^ Conflicting evidence exists as to whether these individuals are a ‘more benign’ group.^9^, ^10^, ^11^, ^12^ Plausibly, adverse cardiovascular outcomes associated with excess adiposity could result from more extreme metabolic phenotypes that arise as part of the sustained obese state.^3^, ^4^, ^8^ Therefore, it is plausible that MHO may be in a transitional state, early in the trajectory of atherosclerosis and CVD. To evaluate this hypothesis further, we evaluate their vascular health by assessing and comparing their arterial stiffness with individuals of healthy body mass index (BMI).

Arterial stiffness is an early manifestation of pathological processes that often precedes the development of hypertension and CVD.^13^ It provides important information on the level of vascular compliance and may detect early vascular damage due to endothelial dysfunction or vascular inflammation, even before the onset of overt disease among individuals with obesity.^13^, ^14^, ^15^ Arterial stiffness has been shown to be associated with adverse cardiovascular outcomes.^13^ The reversibility of arterial stiffness, which has been demonstrated in pharmacological interventions associated with cardiovascular benefit in animal and human studies, provides an opportunity to its use as a proxy for vascular health.^13^, ^16^, ^17^, ^18^, ^19^ Potential insights might inform physicians to consider preventive strategies in individuals with obesity even when typical parameters incorporated into risk scores reflecting metabolic health are absent.^20^, ^21^

Using a large contemporary UK cohort of 162 590 participants, we aimed to assess the independent and joint effects of excess adiposity and metabolic abnormalities on arterial stiffness. Our results will provide insight into whether having obesity alone is sufficient to warrant preventive approaches, or whether one should wait for accompanying metabolic abnormalities to emerge before these individuals are considered to be in poor vascular health, and hence at higher future risk.

## 2 METHODS

### 2.1 Study design

The UK Biobank is a population‐based prospective cohort that recruited over 500 000 participants aged 40–69 years.^22^ Between 2006 and 2010, participants attended 1 of 22 assessment centres across England, Wales and Scotland for functional and physical assessments, and provided blood, urine, and saliva specimens for biochemical and genotype analyses. During the visit, participants also provided sociodemographic and behavioural information through a questionnaire and computer‐assisted interview.

The current study was restricted to the subset of participants with both BMI and arterial stiffness index (ASI) data available at the initial assessment only (March 2006 to December 2010). Participants with CVD, including stroke and myocardial infarction (*n* = 5985), those who were underweight (BMI <18.5 kg/m^2^; *n* = 878), those with ASI measurement failure (*n* = 825) or those with extreme ASI outliers (ASI ≥29.59 m/s; mean ± 5 standard deviations; *n* = 76) were excluded (Figure S1). For those with measurement failure (*n* = 825) due to insufficient waveforms captured, these failures were not influenced by excess adiposity (0.8% of those with normal BMI vs. 0.3% overweight and 0.3% obesity). Considering the cross‐sectional nature of this study, participants with CVD were excluded to account for reverse causality. This study complied with the Declaration of Helsinki, UK Biobank, received ethics approval from the North‐West Multi‐centre Ethics Committee, and all participants provided written informed consent.

### 2.2 Exposure

BMI was calculated using participants' body weight and standing height, which were measured by trained staff during assessment visits. BMI was defined using World Health Organization (WHO) criteria: normal weight (BMI ≥18.5 kg/m^2^ and <25.0 kg/m^2^), overweight (BMI ≥25.0 kg/m^2^ and <30.0 kg/m^2^) and obesity (BMI ≥30.0 kg/m^2^). Abdominal obesity was defined using WHO criteria: waist circumference of ≥102 cm for men and ≥88 cm for women. Ethnicity‐specific waist circumference was not taken into consideration for the classification of abdominal obesity. Metabolic abnormalities were dichotomized based on the presence of hypertension, dyslipidaemia or diabetes status, each given a score of 1 and were summed to create a metabolic score ranging from 0 to 3. Hypertension was defined by having elevated systolic blood pressure (≥140 mmHg) or diastolic blood pressure (≥90 mmHg),^23^ or taking antihypertensive medications. Diabetes was defined by having elevated fasting plasma glucose (≥126 mg/dL or 7.0 mmol/L) or random blood glucose (≥200 mg/dL or 11.1 mmol/L), elevated glycated haemoglobin (HbA1C; ≥48 mmol/mol or 6.5%)^24^ or taking diabetes medications (insulin or oral glucose lowering medications). Dyslipidaemia was defined by having low fasting or non‐fasting high‐density lipoprotein (HDL‐C; ≤1.0 mmol/L for men, ≤1.3 mmol/L for women), or elevated triglycerides (≥2.0 mmol/L),^25^ or taking statins or other lipid lowering agents. HDL‐C and triglycerides were used for the classification of dyslipidaemia because they are known to associate with insulin resistance and metabolic traits.^26^, ^27^

Participants were stratified into 6 mutually exclusive groups, based on metabolic health (yes/no), and BMI (normal, overweight, obesity). Six metabolic categories were developed (Table S1). Metabolically healthy normal weight (MHN) refers to individuals with normal BMI and no metabolic abnormality, while metabolically unhealthy normal weight (MUN) was defined by having normal BMI with ≥1 metabolic abnormality. Metabolically healthy overweight (MHOW) was defined by meeting the BMI criteria for overweight and has no metabolic abnormality, while metabolically unhealthy overweight (MUOW) includes individuals with overweight and ≥1 metabolic abnormality. Conversely, metabolically healthy obesity (MHO) was defined by meeting the BMI criteria for obesity and has no metabolic abnormality, while metabolically unhealthy obesity (MUO) includes individuals with obesity and ≥1 metabolic abnormality.

### 2.3 Outcomes

The primary outcome of interest was the severity of ASI, which provides an indirect estimate of large artery stiffness measured using a non‐invasive technique. Pulse waveform was recorded by clipping a photoplethysmography transducer (PulseTrace PCA2™, CareFusion, USA) to a rested finger or thumb of participants over a period of 10–15 s. The carotid‐to‐femoral pulse transit time was estimated from the volume of dicrotic pulse waveform in the finger as the time difference between a forward compound of waveform travelling through the arterial tree in the lower body and a reflected compound of waveform back to the finger. ASI was calculated by dividing the participants' standing height by the pulse transit time and was expressed as meters per second (m/s). Extreme outlier ASI values were defined as mean ± 5 × standard deviations (SDs) and were excluded from the analyses.

The secondary outcome of interest is the prevalence of arterial stiffening, stratified by BMI and metabolic categories. A threshold value of 10 m/s was used to define arterial stiffening as ASI beyond this value is a significant independent predictor of cardiovascular mortality.^28^

### 2.4 Covariates

Factors known to be associated with BMI phenotypes, metabolic abnormalities and ASI were included in the analysis as confounders. These variables included participants' age, sex (male vs. female), ethnicity (White, Black, Asian, Mixed, Others), self‐reported smoking status (active smoker, ex‐smoker, never‐smoker) and Townsend deprivation quintiles. The Townsend deprivation indices, expressed as quintiles in this study, are area‐level material metrics derived from income, employment and overcrowding, determined by participants' postcode of residence.

### 2.5 Statistical analysis

Descriptive characteristics by BMI phenotypes were presented as means with standard deviation (SD) for normally distributed data, median with 25th and 75th percentiles for skewed data, and frequencies with percentages for categorical data. The characteristics were compared between BMI phenotypes using one‐way analysis of variance (ANOVA) for continuous outcomes or *χ* ^2^ test for categorical data.

Among the six metabolic categories (MHN, MUN, MHOW, MUOW, MHO and MUO), the severity of arterial stiffness was evaluated by multivariate‐adjusted linear regression. The analysis was adjusted for the following covariates: age, sex, smoking status, ethnicity and Townsend deprivation quintiles. Multicollinearity between variables was checked with variance inflation factors (VIFs), using the R package ‘mctest’, where VIF greater than 4 was considered to indicate multicollinearity. Logistic regression was performed to examine the prevalence of arterial stiffening across the six metabolic categories and was adjusted for similar covariates as described above.

Multiple sensitivity analyses were performed to assess the robustness of the primary outcome. A sensitivity analysis was performed to assess for the potential effect of abdominal obesity on arterial stiffness among the six metabolic categories. Another sensitivity analysis was performed by splitting the six metabolic categories into categories of BMI phenotypes with a cumulative score of metabolic abnormality (e.g. normal weight with 1 metabolic abnormality). Moreover, further sensitivity analyses were performed by adding physical activity, alcohol intake consumption and daily sleep duration as covariates into the main regression models in a stepwise manner. Another sensitivity analysis was performed by adjusting the linear regression for biomarkers of metabolic health profile, including systolic blood pressure, HbA1C, HDL‐C and triglyceride level. Further sensitivity analysis was performed to assess for possible interactions between metabolic score and BMI phenotypes. Wilcoxon rank sum test was performed to assess the *p* value for linear trend of the relationship between ASI and metabolic score within respective BMI categories. Missingness was scarce (≤0.2% in all variables of regression models) and was assumed to be at random. All analyses were analysed using R version 4.0.2, and a 95% confidence interval (CI) was calculated for all outcomes.

## 3 RESULTS

Descriptive statistics of participants by BMI phenotypes are presented in Table 1. A total of 162 590 participants were included, with a mean age of 57 years (SD = 8). Among the participants, 55.0% (*n* = 89 437) were female, one third had university qualifications (*n* = 54 916) and 55.7% (*n* = 90 363) were never‐smokers.

**TABLE 1:** Baseline characteristics of participants stratified by body mass index.

Characteristics	All (*n* = 162 590)	Body mass index		
		Normal (*n* = 53 835)	Overweight (*n* = 69 072)	Obesity (*n* = 39 683)
Age, mean (SD)	57 (8)	56 (8)	57 (8)	57 (8)
Sex, *n* (%)				
Male	73 153 (45.0)	18 668 (34.7)	36 234 (52.5)	18 251 (46.0)
Female	89 437 (55.0)	35 167 (65.3)	32 838 (47.5)	21 432 (54.0)
Ethnicity^a^, *n* (%)				
White	147 543 (90.9)	49 419 (91.9)	62 655 (90.9)	35 469 (89.6)
Black	4360 (2.7)	794 (1.5)	1808 (2.6)	1758 (4.4)
Asian	6265 (3.9)	2223 (4.1)	2788 (4.0)	1254 (3.2)
Mixed	1253 (0.8)	471 (0.9)	481 (0.7)	301 (0.8)
Others	2099 (1.3)	604 (1.1)	900 (1.3)	595 (1.5)
Abdominal obesity^a^, *n* (%)	54 935 (33.8)	1228 (2.3)	18 891 (27.4)	34 816 (87.8)
Education^a^, *n* (%)				
Has college or university qualifications	54 916 (33.8)	22 011 (40.9)	22 487 (32.6)	10 418 (26.3)
Others	107 383 (66.0)	31 745 (59.0)	46 467 (67.3)	29 171 (73.5)
Region of residences^a^, *n* (%)				
Urban	140 609 (87.3)	46 334 (87.0)	59 462 (87.0)	34 813 (88.5)
Regional and rural areas	20 370 (12.7)	6944 (13.0)	8911 (13.0)	4515 (11.5)
Townsend deprivation quintiles^a^, *n* (%)				
Q1 (least deprived)	32 642 (20.1)	11 477 (21.4)	144 449 (20.9)	6716 (17.0)
Q2	32 434 (20.0)	11 063 (20.6)	14 233 (20.6)	7138 (18.0)
Q3	32 414 (20.0)	10 696 (19.9)	14 059 (20.4)	7659 (19.3)
Q4	32 465 (20.0)	10 907 (20.3)	13 447 (19.5)	8111 (20.5)
Q5 (most deprived)	32 372 (19.9)	9604 (17.9)	12 782 (18.5)	9986 (25.2)
Smoking status^a^, *n* (%)				
Never‐smoker	90 363 (55.7)	32 082 (59.7)	37 540 (54.4)	20 741 (52.4)
Ex‐smoker	55 173 (34.0)	15 812 (29.4)	24 362 (35.3)	14 999 (37.9)
Current smoker	16 054 (9.9)	5696 (10.6)	6734 (9.8)	3624 (9.2)
Physical activity^a^				
Summed MET minutes per week for all activity, mean (SD)	2735 (2737)	2941 (2769)	2767 (2749)	2380 (2632)
Sleep duration^a^ (hours per day), mean (SD)	7 (1)	7 (1)	7 (1)	7 (1)
Alcohol intake frequency^a^, *n* (%)				
Never	13 980 (8.6)	4307 (8.0)	5428 (7.9)	4245 (10.7)
Special occasion only	19 822 (12.2)	5583 (10.4)	7713 (11.2)	6526 (16.5)
One to three times a month	18 439 (11.4)	5618 (10.5)	7388 (10.7)	5433 (13.7)
Once or twice a week	40 805 (25.1)	13 301 (24.7)	10 011 (25.3)	17 493 (25.4)
Three or four times a week	36 231 (22.3)	12 806 (23.8)	16 162 (23.4)	7263 (18.3)
Daily or almost daily	32 779 (20.2)	12 069 (22.5)	14 667 (21.3)	6043 (15.3)
Fasting blood specimens^a^, *n* (%)	4254 (2.6)	1048 (1.9)	1771 (2.6)	1435 (3.6)
Hypertension, *n* (%)	86 407 (53.1)	20 602 (38.3)	38 146 (55.2)	27 659 (69.7)
Diabetes, *n* (%)	8041 (4.9)	909 (1.7)	2791 (4.0)	4341 (10.9)
Dyslipidaemia, *n* (%)	69 797 (42.9)	13 111 (24.4)	31 818 (46.1)	24 868 (62.7)
Metabolic score^b^, *n* (%)				
0	50 710 (31.2)	26 521 (49.3)	18 865 (27.3)	5324 (13.4)
1	65 197 (40.1)	20 485 (38.1)	29 517 (42.7)	15 195 (38.3)
2	41 001 (25.2)	6350 (11.8)	18 832 (27.3)	15 819 (39.9)
3	5682 (3.5)	479 (0.9)	1858 (2.7)	3345 (8.4)
Arterial stiffness index (m/s), mean (SD)	9.28 (3.05)	8.71 (2.99)	9.47 (3.06)	9.74 (3.00)
Prevalence of arterial stiffening (≥ 10 m/s), *n* (%)	62 001 (38.1)	16 159 (30.0)	28 279 (40.9)	17 563 (44.3)

^a^ Missing data: ethnicity, education qualifications, smoking status (291; 0.2%); abdominal obesity (36; 0.02%); region of residences (1659; 1.0%); Townsend deprivation quintiles (263; 0.2%); fasting status for blood specimens (88; 0.05%); sleep duration (1352; 0.8%); alcohol intake frequency (534; 0.3%); physical activity (30 401; 18.7%).

^b^ Metabolic score is defined as the cumulative number of metabolic abnormalities in a participant, including hypertension, diabetes and dyslipidaemia.

With respect to BMI, 33.1% (*n* = 53 835) participants were of normal BMI, 42.5% (*n* = 69 072) were overweight and 24.4% (*n* = 39 683) were obese. One quarter of participants with overweight (*n* = 18 891) had abdominal obesity, compared to 87.8% of participants with obesity (*n* = 34 816). Those with normal BMI were more likely to be of female sex, have college or university qualifications, and higher levels of physical activity. The group with obesity had more participants of Black ethnicity and were more likely to live in more deprived areas and have multiple metabolic abnormalities.

Table 2 presents participants' characteristics by metabolic health profile. Metabolically healthy participants (i.e. those with no metabolic abnormality) were younger, were more likely to be of female sex, have normal BMI and have college or university qualifications. Three quarters of participants with metabolic abnormalities were overweight or obese. There was no difference in deprivation quintiles between the cohorts, stratified by the metabolic health status.

**TABLE 2:** Baseline participants characteristics, stratified by metabolic health profile.

Characteristics	All (*n* = 162 590)	Metabolic health profile	
		Healthy (*n* = 50 710)	Unhealthy (*n* = 111 880)
Age, mean (SD)	57 (8)	54 (8)	58 (8)
Sex, *n* (%)			
Male	73 153 (45.0)	17 445 (34.4)	55 708 (49.8)
Female	89 437 (55.0)	33 265 (65.6)	56 172 (50.2)
Ethnicity^a^, *n* (%)			
White	147 543 (90.9)	46 486 (92.2)	101 057 (90.9)
Black	4360 (2.7)	1337 (2.7)	3023 (2.7)
Asian	6265 (3.9)	1443 (2.9)	4822 (4.4)
Mixed	1253 (0.8)	505 (1.0)	748 (0.7)
Others	2099 (1.3)	636 (1.2)	1463 (1.3)
Body mass index, *n* (%)			
Normal	53 835 (33.1)	26 251 (52.3)	27 314 (24.4)
Overweight	69 072 (42.5)	18 865 (37.2)	50 207 (44.9)
Obesity	39 683 (24.4)	5324 (10.5)	34 359 (30.7)
Abdominal obesity^a^, *n* (%)	54 935 (33.8)	8663 (17.1)	46 272 (41.4)
Education^a^, *n* (%)			
Has college or university qualifications	54 916 (33.8)	20 719 (40.9)	34 197 (30.6)
Others	107 383 (66.0)	29 909 (59.1)	77 474 (69.4)
Region of residences^a^, *n* (%)			
Urban	140 609 (87.3)	43 963 (87.8)	96 646 (87.1)
Regional and rural areas	20 370 (12.7)	6111 (12.2)	14 259 (12.9)
Townsend deprivation quintiles^a^, *n* (%)			
Q1 (least deprived)	32 642 (20.1)	10 341 (20.4)	22 301 (20.0)
Q2	32 434 (20.0)	10 109 (20.0)	22 325 (20.0)
Q3	32 414 (20.0)	10 177 (20.1)	22 237 (19.9)
Q4	32 465 (20.0)	10 404 (20.5)	22 061 (19.7)
Q5 (most deprived)	32 372 (19.9)	9600 (19.0)	22 772 (20.4)
Smoking status^a^, *n* (%)			
Never‐smoker	90 363 (55.7)	30 101 (59.5)	60 262 (54.0)
Ex‐smoker	55 173 (34.0)	15 458 (30.5)	39 715 (35.6
Current smoker	16 054 (9.9)	4904 (9.7)	11 150 (10.0)
Physical activity^a^			
Summed MET minutes per week for all activity, mean (SD)	2735 (2737)	2846 (2743)	2683 (2733)
Sleep duration^a^ (hours per day), mean (SD)	7 (1)	7 (1)	7 (1)
Alcohol intake frequency^a^, *n* (%)			
Never	13 980 (8.6)	3599 (7.1)	10 381 (9.3)
Special occasion only	19 822 (12.2)	5341 (10.5)	14 481 (13.0)
One to three times a month	18 439 (11.4)	5873 (11.6)	12 566 (11.3)
Once or twice a week	40 805 (25.1)	13 571 (26.8)	27 234 (24.4)
Three or four times a week	36 231 (22.3)	12 339 (24.4)	23 892 (21.4)
Daily or almost daily	32 779 (20.2)	9844 (19.4)	22 935 (20.5)
Arterial stiffness index (m/s), mean (SD)	9.28 (3.05)	8.54 (2.71)	9.62 (3.14)
Prevalence of arterial stiffening (≥ 10 m/s), *n* (%)	62 001 (38.1)	14 038 (27.7)	47 963 (42.9)

^a^ Missing data: ethnicity, education qualifications, smoking status (291; 0.2%); abdominal obesity (36; 0.02%); region of residences (1659; 1.0%); Townsend deprivation quintiles (263; 0.2%); sleep duration (1352; 0.8%); alcohol intake frequency (534; 0.3%); physical activity (30 401; 18.7%).

### 3.1 Arterial stiffness severity

Participants with overweight and obesity had higher mean ASI (9.47 and 9.74 m/s, respectively, Table 1) than those with normal weight (8.71 m/s; *p* < 0.001). Participants with metabolic abnormalities (mean 9.62 m/s, Table 2) had more severe ASI than those with no metabolic abnormalities (mean 8.54 m/s; *p* < 0.001).

**FIGURE 1:**
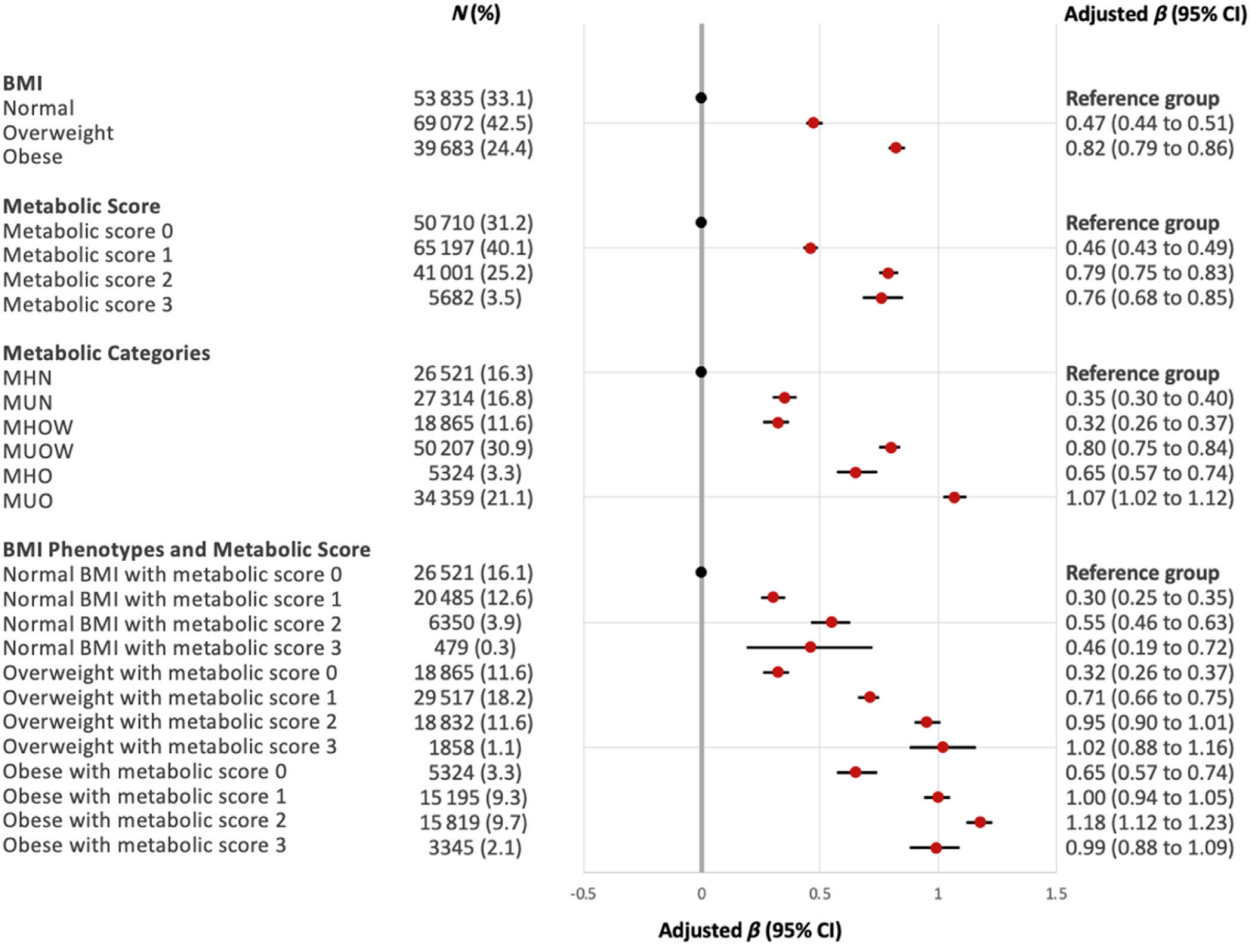
Forest plot comparing the severity of arterial stiffness index, stratified by BMI phenotypes and metabolic abnormalities. Regression coefficients (β) were estimated using linear regression and were adjusted age, sex, smoking, ethnicity and Townsend deprivation quintiles. Variance inflation factor (VIF) was less than 4 for all analysis. BMI, body mass index; CI, confidence interval; MHN, metabolically healthy normal weight; MUN, metabolically unhealthy normal weight; MHOW, metabolically healthy overweight; MUOW, metabolically unhealthy overweight; MHO, metabolically healthy obesity; MUO, metabolically unhealthy obesity.

Participants with normal BMI and no metabolic abnormalities (MHN; reference group) had less severe ASI than metabolically healthy participants with overweight (adjusted β‐coefficient [β] 0.32; 95% CI 0.26 to 0.37; *p* < 0.001; Figure 1; Table S2) and obesity (β 0.65; 95% CI 0.57 to 0.74; *p* < 0.001). Compared to participants with no metabolic abnormality, participants with higher metabolic score had more severe ASI: metabolic score 1 (β 0.46; 95% CI 0.43 to 0.49); 2 (β 0.79; 95% CI 0.75 to 0.83); 3 (β 0.76; 95% CI 0.68 to 0.85; *p* < 0.001 for all comparisons).

Participants with obesity and metabolic abnormalities (MUO) had the highest ASI (β 1.07; 95% CI 1.02 to 1.12) compared with MHN among all metabolic categories after adjusting for covariates (Figure 1; Table S2). Participants with overweight and metabolic abnormalities had more severe ASI (β 0.80; 95% CI 0.75 to 0.84) than metabolically healthy participants with overweight and obesity, when compared to the reference group. Metabolically unhealthy participants with normal BMI (β 0.35; 95% CI 0.30 to 0.40) had comparable ASI to metabolically healthy participants with overweight (β 0.32; 95% CI 0.26 to 0.37; *p* < 0.001 for all metabolic categories vs. reference group). Assessment of multicollinearity among variables revealed no violations (VIF <4 for all analysis).

Sensitivity analysis showed in general a positive association between metabolic score and ASI within each subgroup of BMI phenotypes (Figure 1; Table S2), although this was less clear for individuals of normal BMI and obesity with three metabolic abnormalities which may be masked by insufficient power (*N* = 479, 0.3%, and *N* = 3345, 2.1% participants, respectively). However, the *p* value for linear trend was <0.001 for all trends, showing a positive association between ASI and metabolic score within their respective BMI categories.

Further sensitivity analysis with adjustments for physical activity, alcohol intake frequency and sleep duration did not attenuate the association of ASI with BMI phenotypes and metabolic health in all categories (Table S3). After adjusting for conventional cardiometabolic health markers (e.g. systolic blood pressure, high‐density lipoprotein, triglycerides, HbA1C), the ASI of all metabolically unhealthy individuals was attenuated (Table S4). Among individuals with obesity, the severity of ASI varies with metabolic score, but this interaction was not observed among individuals with normal BMI or overweight (Table S5).

**FIGURE 2:**
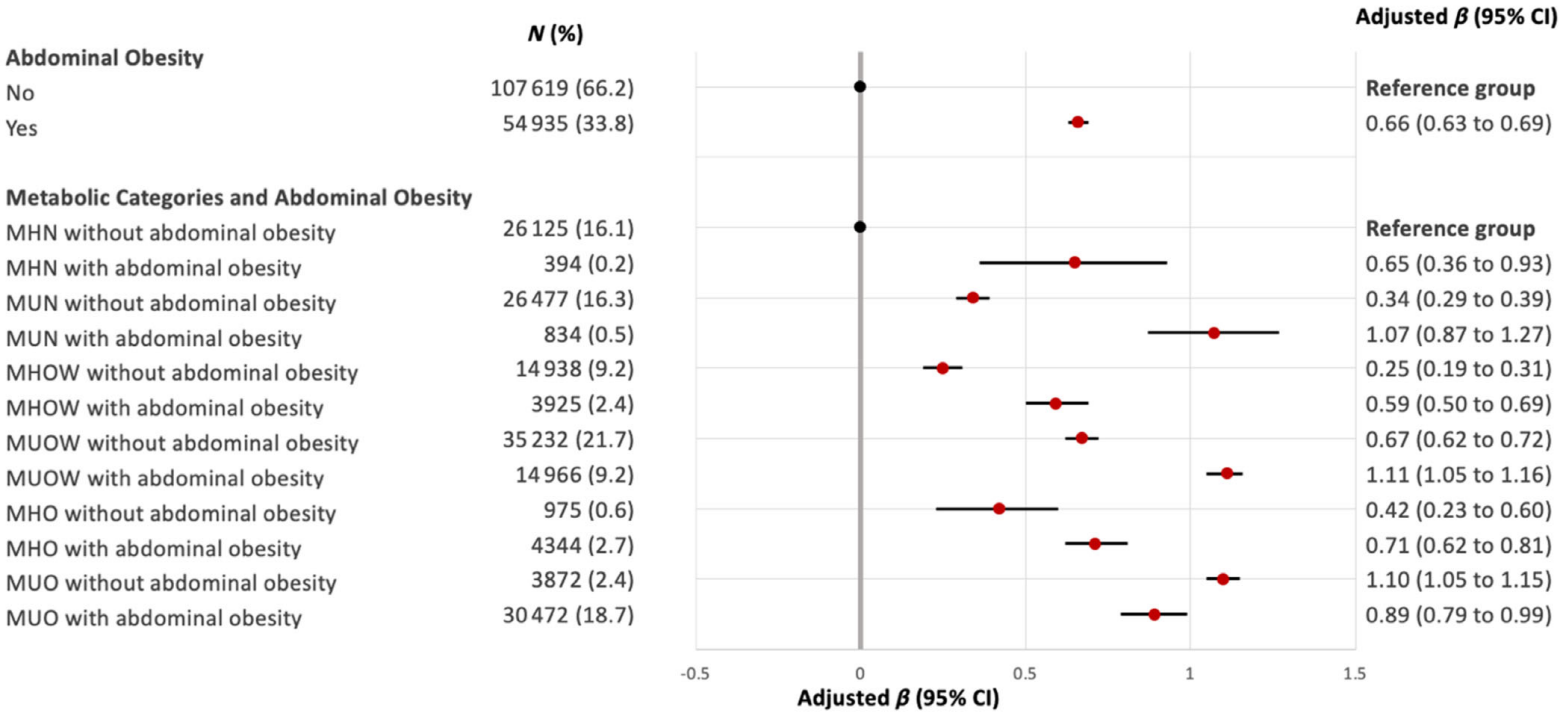
Forest plot comparing the severity of arterial stiffness index, stratified by abdominal obesity, BMI phenotypes and metabolic abnormalities. Regression coefficients (β) were estimated using linear regression and were adjusted age, sex, smoking, ethnicity and Townsend deprivation quintiles. Variance inflation factor (VIF) was less than 4 for all analysis. BMI, body mass index; CI, confidence interval; MHN, metabolically healthy normal weight; MUN, metabolically unhealthy normal weight; MHOW, metabolically healthy overweight; MUOW, metabolically unhealthy overweight; MHO, metabolically healthy obesity; MUO, metabolically unhealthy obesity.

Participants with abdominal obesity had higher ASI than their counterparts without abdominal obesity, within their respective metabolic categories (Figure 2; Table S6). This relationship persisted even among those with normal BMI: MHN with abdominal obesity (β 0.65 vs. reference group), and MUN with abdominal obesity (β 1.07 vs. 0.34 in MUN without abdominal obesity).

### 3.2 Arterial stiffening

Prevalence of arterial stiffening was higher among individuals with overweight (40.9%) and obesity (44.3%) compared to those with normal BMI (30.0%; Table 1). Participants with metabolic abnormalities had higher prevalence of arterial stiffening than those without metabolic abnormalities (42.9% vs. 27.7%; Table 2).

**FIGURE 3:**
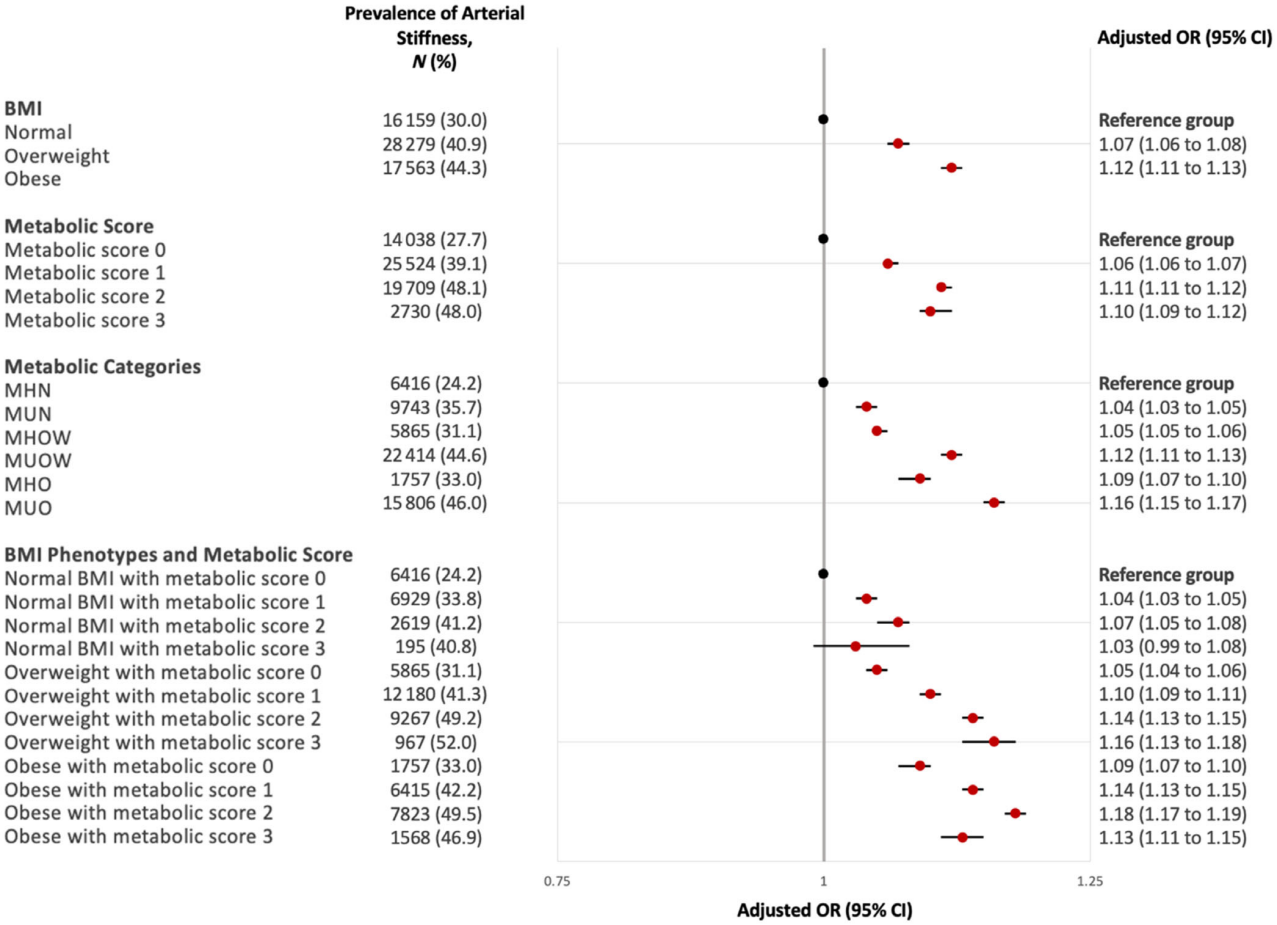
Forest plot showing the odds of arterial stiffening, stratified by BMI phenotypes and metabolic abnormalities. Arterial stiffening was defined as having arterial stiffness index of 10 m/s and above. Odds ratios were estimated using logistic regression and were adjusted age, sex, smoking, ethnicity and Townsend deprivation quintiles. BMI, body mass index; CI, confidence interval; MHN, metabolically healthy normal weight; MUN, metabolically unhealthy normal weight; MHOW, metabolically healthy overweight; MUOW, metabolically unhealthy overweight; MHO, metabolically healthy obesity; MUO, metabolically unhealthy obesity; OR, odd ratio.

Compared with participants with normal BMI and no metabolic abnormality, the odds of arterial stiffening were higher among metabolically healthy participants with overweight (odds ratio (OR) 1.05; 95% CI 1.04 to 1.05; *p* < 0.001) and obesity (OR 1.09; 95% CI 1.07 to 1.10; *p* < 0.001; Figure 3 and Table S7). Participants with higher metabolic score had higher odds of arterial stiffening compared to those with no metabolic abnormality: metabolic score 1 (OR 1.06; 95% CI 1.06 to 1.07); 2 (OR 1.11; 95% CI 1.11 to 1.12); 3 (OR 1.10; 95% CI 1.09 to 1.12; *p* < 0.001 for all comparisons).

Participants with obesity and metabolic abnormalities had the highest odds of arterial stiffening compared with MHN (reference group) among all metabolic categories (OR 1.16; 95% CI 1.15 to 1.17). Participants with overweight and metabolic abnormalities (OR 1.12; 95% CI 1.11 to 1.13) had higher odds of arterial stiffening than metabolically healthy participants with overweight and obesity when compared to the reference group. The odds of arterial stiffening were similar between metabolically unhealthy participants with normal BMI (OR 1.04; 95% CI 1.03 to 1.05) and metabolically healthy participants with overweight (OR 1.05; 95% CI 1.04 to 1.05; *p* < 0.001 for all metabolic categories vs. reference group).

Based on the sensitivity analysis, the odds of arterial stiffening increased with metabolic score (Figure 3; Table S7) within the subgroup of BMI phenotypes, except for individuals of normal BMI and obesity with 3 metabolic abnormalities, possibly due to insufficient power in these groups (*N* = 479 and *N* = 3345, respectively).

## 4 DISCUSSION

This study showed that individuals with overweight or obesity have worse arterial stiffness (a proxy for vascular health) even in the absence of metabolic abnormalities, compared to individuals of healthy normal BMI without metabolic abnormalities. These effects occur in a graded fashion with worsening BMI associated with worse arterial stiffness and a greater likelihood of the dichotomous cutoff used to define arterial stiffness. Abdominal obesity was also associated with worse arterial stiffness within each BMI category, and these associations were independent of age, sex, smoking status, and socioeconomic factors. Arterial stiffness and the prevalence of arterial stiffening also increased with worsening metabolic score in all individuals within each BMI category. Approximately half of the individuals with normal BMI had ≥1 metabolic abnormality, and this was associated with higher arterial stiffness compared to metabolically healthy individuals with normal BMI. This phenotype exhibited similar arterial stiffness as individuals who are overweight but do not have metabolic abnormalities. Arterial stiffness was most severe when both obesity and metabolic disturbances were present.

Our findings highlight the independent adverse effects of the overweight and obesity phenotypes on vascular health as assessed by arterial stiffness, even prior to the onset of significant metabolic abnormalities. Our findings of early manifestation of adverse vascular health measures have practical implications for determining the timing of preventive strategies for individuals with overweight and obesity. Using existing risk calculators, these individuals would cross specific risk parameters only when additional measures of metabolic disturbance typically included in risk scores become elevated.^20^, ^21^, ^29^ Typically, at that stage, pharmacotherapy such as cholesterol‐lowering, blood pressure lowering and glucose‐lowering medications may be recommended.^29^ The present findings support the notion that among individuals who are overweight or obese without evidence of metabolic abnormalities, it may be reasonable to perhaps consider typical primary prevention pharmacotherapy such as lipid modification therapies, as part of shared decision making, in addition to lifestyle and pharmacotherapy to achieve weight loss. A relevant consideration given the increasing prevalence of obesity, whereby 40% of the world population and over two‐thirds of Europeans are living with overweight or obesity.^30^ Although the impact of obesity on arterial stiffness is multifactorial, the absence of abnormal metabolic phenotype among MHOW and MHO individuals suggests that these findings may be mainly driven by obesity‐related factors such as endothelial dysfunction and low‐grade vascular inflammation.^14^, ^15^ This effect may be mediated by the hormone leptin, as higher levels of leptin may increase oxidative stress in endothelial cells and promote vascular smooth muscle cell proliferation and angiogenesis.^31^, ^32^, ^33^ Besides, obesity can lead to a chronic state of low‐grade systemic inflammation due to higher circulating levels of several inflammatory cytokines, including interleukin‐6 and tumour necrosis factor.^15^, ^34^ These factors may have contributed to the obesity‐related arterial stiffness observed in our participants before the onset of overt metabolic diseases.

Although abnormal metabolic phenotype is associated with higher cardiovascular risk, having a normal metabolic profile may not protect an individual from reduced vascular compliance when they are overweight or obese as compared with a normal BMI.^2^ A meta‐analysis conducted by Petersen et al. found that modest weight reduction can reduce arterial stiffness, but their findings should be interpreted with caution as some studies included in their meta‐analysis were either single‐arm or non‐randomized trials.^35^ However, our findings of independent adverse effects associated with obesity even after adjusting for physical activity levels, alcohol consumption and sleep, highlight the importance of BMI phenotypes on arterial stiffness suggest that the findings by Petersen et al. are probably justified.^35^ Therefore, it is important to recommend weight‐loss interventions to individuals with overweight and obesity, and to prevent normal weight individuals from gaining excess weight. Weight loss should start with healthy lifestyle modifications, which are cost‐effective strategies and feasible even in resource‐limited settings, followed by pharmacotherapy and bariatric surgery if the former fails.^36^, ^37^ Moreover, our observation of an interaction between obesity and the presence of two or more metabolic abnormalities on ASI may indicate that a higher risk phenotype that could potentially require or benefit more from weight loss therapies, but this requires further evaluation by clinical trials.

Only three cohort studies had previously assessed the arterial stiffness of MHO individuals, but the analyses have several limitations.^9^, ^10^, ^11^ Consistent with our findings, these studies demonstrated that metabolically unhealthy individuals had more severe arterial stiffness, irrespective of BMI phenotypes.^9^, ^10^, ^11^ However, the authors reported statistically insignificant results for those with MHO phenotype, which contrasts previous systematic reviews that highlight higher risk of cardiovascular diseases among MHO individuals.^9^, ^10^, ^11^, ^12^ Additionally, these studies had smaller study populations and recruited Asian participants only (*N* = 2076, 6220 and 22 153, respectively), as opposed to our multiethnic population with 162 590 participants.^9^, ^10^, ^11^ The study populations were also younger (36–48 years old), and had a lower short‐term risk of atherosclerotic cardiovascular diseases.^9^, ^38^ Yuan et al. employed a higher cut‐off (≥14 m/s) for arterial stiffness, as opposed to our threshold which was set at 10 m/s, in accordance with European Society of Hypertension and European Society of Cardiology guidelines.^9^, ^28^, ^39^ The higher cutoff was proposed from an assessment of a Japanese population based on the Framingham risk score, but Japanese populations are known to have lower CVD risk than other populations.^40^ The studies conducted by Lin et al. and Yuan et al. employed a lower cut‐off for obesity (BMI ≥25.0 kg/m^2^), but this is appropriate for Asian population according to WHO expert consultation.^9^, ^10^, ^41^ Still, a different definition of ‘metabolically healthy’ phenotype (metabolic score ≤2) was used in these studies.^9^, ^10^, ^11^ Despite these potential limitations, unadjusted and initial adjusted analysis (age and sex only) conducted by Yuan et al. suggested significantly higher odds of arterial stiffening in MHO individuals than MHN individuals. These associations attenuated to the null after adjusting for several other confounders, including BMI and cardiometabolic markers.^9^ This may result from model overfitting as the analysis was adjusted for several covariates that were part of exposures (e.g. BMI, metabolic abnormalities) and other covariates that are highly correlated.^9^ In that study, Yuan et al. also adjusted the models for multiple levels of similar exposure variable (e.g. history of dyslipidaemia, total cholesterol, triglycerides, HDL‐C, LDL).^9^, ^10^

In the present study, we have selected and adjusted for important confounders only, and no multicollinearity was identified. Missing data of all variables included in the regression models was less than 0.2%. Our study has also shown that individuals who are overweight and obese, with or without metabolic abnormalities, had more severe arterial stiffness than normal weight counterparts, even after adjusting for traditional cardiometabolic risk factors (metabolic health profile) (Table S4). Moreover, whilst BMI and metabolic health are each independently associated with ASI, we observed an interaction among individuals with obesity who had 2 or more metabolic abnormalities, suggesting a multiplicative effect and identifying a group potentially at even greater risk (Table S5). The large sample size of the present study provides hitherto greater statistical power than previously feasible. This allowed sufficient precision in sensitivity analyses to examine all individuals stratified by different metabolic score, which may reflect different definitions of the ‘metabolically healthy’ phenotype used in different clinical contexts.^7^, ^8^

Our study highlights the independent adverse effect of metabolic abnormalities on vascular health and arterial stiffness. This effect was worse in the presence of excess weight. However, having a normal BMI did not protect individuals from developing metabolic abnormalities.^42^ In our study, more than half of individuals with normal BMI had ≥1 metabolic abnormalities, and their vascular health was comparable to individuals who are overweight with no metabolic abnormalities, highlighting the importance of early detection and management of metabolic abnormality among individuals of normal BMI for primary prevention of cardiovascular diseases.

However, this study has several limitations. First, the UK Biobank data may not be completely representative of the UK as only 5.5% of those invited enrolled in the study, hence the data may be subject to healthy volunteer selection bias. Despite a low participation rate, estimates of effect size were found to be consistent with other population‐representative cohorts in the UK.^43^ Another limitation is that the study population is predominantly of White ethnicity (91%); thus, the findings may not be generalisable to other ethnic groups. Although this study has adjusted for major confounders associated with arterial stiffness, residual confounding cannot be eliminated in an observational study. Furthermore, we used ASI as a proxy of vascular health, rather than hard outcomes.

BMI has several advantages as a simple and reproducible surrogate of body fat and measure of obesity, but it cannot distinguish enhanced lean muscle mass from high body fat percentage. Although bioimpedance‐based BMI datasets were available from UK Biobank, calculated BMI was utilised in our study due to its superiority as a predictor of overall adiposity in the general population over bioimpedance analysis based on a large population study (>12 000 subjects) from the National Health and Nutrition Examination Survey.^44^ ASI provides an estimation of large artery stiffness, but previous studies have shown that it correlates well with carotid‐femoral pulse wave velocity (*r* = 0.65), which is a gold‐standard test to measure large artery stiffness.^45^, ^46^, ^47^ Previous Bland–Altman analyses also indicate close agreement in the 95% CI for differences in Z scores between ASI and carotid‐femoral pulse wave velocity.^45^

In addition to medications and prescription codes, we also used clinical and laboratory parameters for the diagnosis of hypertension (blood pressure), diabetes (blood glucose and HbA1C concentration), and dyslipidaemia (serum lipids) to minimise misclassification bias, as a proportion of participants with metabolic abnormalities may be undiagnosed or not receiving treatment in the UK. However, we cannot eliminate potential misclassification bias from medications or prescription codes as a proportion of blood‐pressure lowering medications can be used for other medical conditions, including heart failure, cardiac arrhythmias and anxiety. Another limitation is that the temporal relationship between exposures (BMI and metabolic abnormalities) and outcome (arterial stiffness) was not assessed in this study due to its cross‐sectional nature, and prospective longitudinal studies would strengthen our findings. Finally, this study also cannot eliminate the possibility of misclassification bias from exposure measurements.

In conclusion, excess adiposity and metabolic abnormalities are independently and jointly associated with poor vascular health and arterial stiffness, suggesting that individuals who are overweight and obese with no metabolic abnormalities are not cardiovascular benign groups. Even in the absence of metabolic abnormalities, cardiovascular preventive strategies and weight management strategies should be considered in these individuals as they already have early manifestations of vascular dysfunction. Longitudinal analysis of the progression of arterial stiffness among these individuals over time and their relationship with incident cardiovascular diseases could reinforce these recommendations.

## AUTHOR CONTRIBUTIONS

All authors were involved in the study conceptualisation. R.A. carried out the formal analysis and drafted the manuscript. B.S. and K.K.R. critically revised the manuscript. All the authors gave final approval and agreed to be accountable for all the aspects of work ensuring integrity and accuracy.

## FUNDING INFORMATION

This study was supported by postgraduate student project funding from the School of Public Health, Imperial College London.

## CONFLICT OF INTEREST STATEMENT

K.K.R. reports unrestricted research grants to Imperial College London from Amgen, Daiichi Sankyo, Regeneron, Sanofi, and SC, EC or advisory board honoraria from Novartis, Esperion, Daiichi Sankyo, Abbott, Bayer, Eli Lilly, Silence Therapeutics, CSL Behring, New Amsterdam Pharma, Sanofi, Amgen, Novo Nordisk, BI, Scribe, Vaxxinity, CRISPR, AZ, Kowa, and Cargene, honoraria for CME and non‐CME from Novartis, Novo Nordisk, BI, AZ, Viatris, Daiichi Sankyo, Amgen, and Sanofi, and stock options from PEMI‐31 New Amsterdam Pharma and Scribe Therapeutics. The other authors have no disclosures.

### PEER REVIEW

The peer review history for this article is available at https://www.webofscience.com/api/gateway/wos/peer‐review/10.1111/dom.16090.

## Supporting information


**Data S1.** Supporting Information.

## Data Availability

Data may be obtained from a third party and are not publicly available. This research was conducted using the UK Biobank resource under access application 104072. UK Biobank will make the data available to all bona fide researchers for all types of health‐related research that is in the public interest, without preferential or exclusive access for any persons. All researchers will be subject to the same application process and approval criteria as specified by UK Biobank. For more details on the access procedure, see the UK Biobank website: http://www.ukbiobank.ac.uk/register-apply.
