# Supplementary material for: Individual and joint associations of obesity and metabolic health parameters on arterial stiffness: Evidence from the UK Biobank
Source: Diabetes Obes Metab. 2024 Nov 25;27(2):899–910. doi: 10.1111/dom.16090 (PMC11701184; doi:10.1111/dom.16090)
Supplement: Supplementary file 1 — Data S1. Supporting Information. [file DOM-27-899-s001.docx]

**Supplementary Materials**


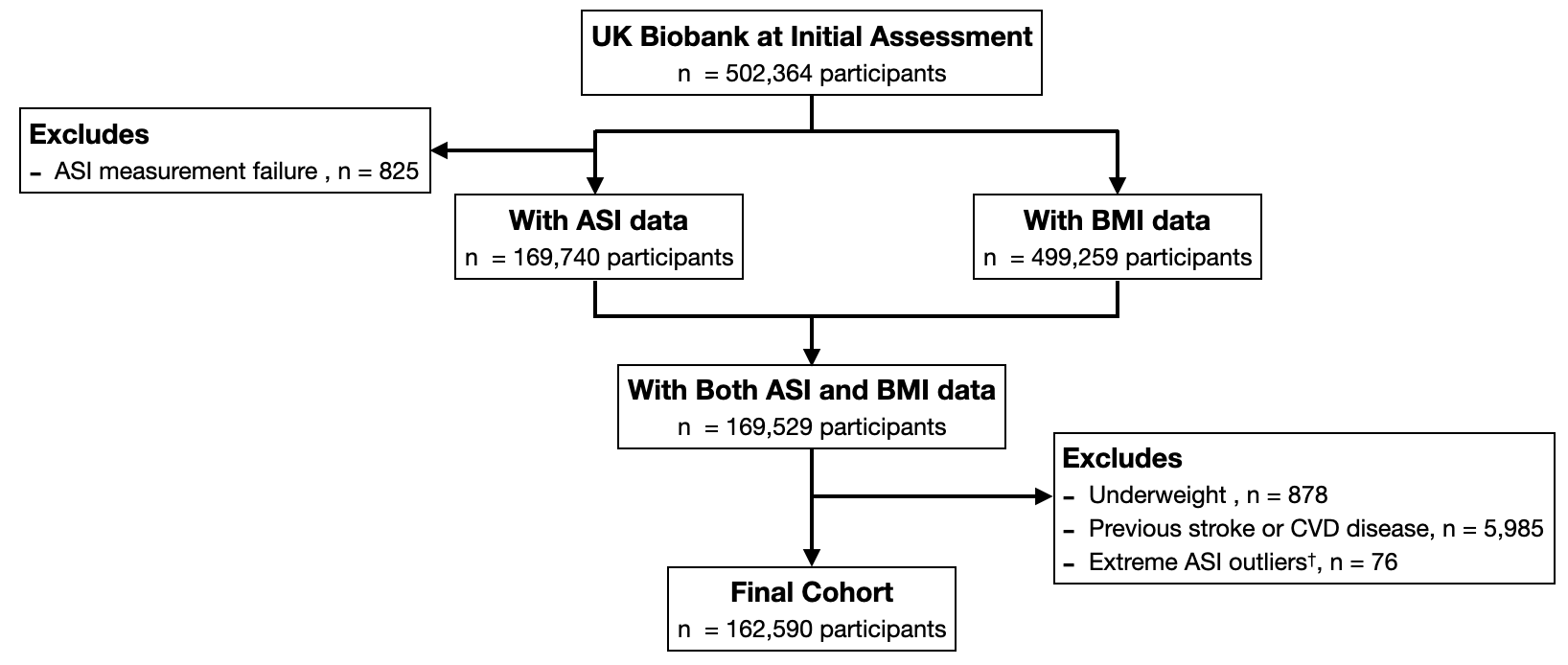


**Figure S1:** Flowchart of participant selection for analysis

^†^Outliers were defined as mean +/- 5 standard deviations (ASI ≥ 29.59 m/s).

**Table S1.** Summary of metabolic health categories within each BMI phenotype.

| **Metabolic category** | **Abbreviation** | **BMI** | **Metabolic score** |
| --- | --- | --- | --- |
| Metabolically healthy normal weight | MHN | Normal | 0 |
| Metabolically unhealthy normal weight | MUN | Normal | ≥1 |
| Metabolically healthy overweight | MHOW | Overweight | 0 |
| Metabolically unhealthy overweight | MUOW | Overweight | ≥1 |
| Metabolically healthy obesity | MHO | Obesity | 0 |
| Metabolically unhealthy obesity | MUO | Obesity | ≥1 |

**Table S2** Relationship between arterial stiffness index, BMI phenotypes, and metabolic score.

| **Clinical Variables** | **N (%)** | **Unadjusted** | | **Adjusted^†^** | |
| --- | --- | --- | --- | --- | --- |
|  |  | **β (95% CI)** | **p-value** | **β (95% CI)** | **p-value** |
| **BMI** | | | | | |
| Normal | 53835 (33.1) | Ref |  | Ref |  |
| Overweight | 69072 (42.5) | 0.76  (0.72 to 0.79) | < 0.001 | 0.47^*^  (0.44 to 0.51) | < 0.001 |
| Obesity | 39683 (24.4) | 1.03  (0.99 to 1.07) | < 0.001 | 0.82**^*^**  (0.79 to 0.86) | < 0.001 |
| **Metabolic Score** | | | | | |
| 0 | 50710 (31.2) | Ref |  | Ref |  |
| 1 | 65197 (40.1) | 0.82  (0.78 to 0.85) | < 0.001 | 0.46**^*^**  (0.43 to 0.49) | < 0.001 |
| 2 | 41001 (25.2) | 1.43  (1.39 to 1.47) | < 0.001 | 0.79**^*^**  (0.75 to 0.83) | < 0.001 |
| 3 | 5682 (3.5) | 1.50  (1.42 to 1.58) | < 0.001 | 0.76**^*^**  (0.68 to 0.85) | < 0.001 |
| **Metabolic Categories**^¥^ | | | | | |
| MHN | 26521 (16.3) | Ref |  | Ref |  |
| MUN | 27314 (16.8) | 0.84  (0.78 to 0.89) | < 0.001 | 0.35**^*^**  (0.30 to 0.40) | < 0.001 |
| MHOW | 18865 (11.6) | 0.49  (0.43 to 0.55) | < 0.001 | 0.32**^*^**  (0.26 to 0.37) | < 0.001 |
| MUOW | 50207 (30.9) | 1.44  (1.39 to 1.48) | < 0.001 | 0.80**^*^**  (0.75 to 0.84) | < 0.001 |
| MHO | 5324 (3.3) | 0.69  (0.60 to 0.77) | < 0.001 | 0.65**^*^**  (0.57 to 0.74) | < 0.001 |
| MUO | 34359 (21.1) | 1.57  (1.52 to 1.62) | < 0.001 | 1.07**^*^**  (1.02 to 1.12) | < 0.001 |
| **BMI Phenotypes and Metabolic Score** | | | | | |
| Normal, 0 | 26521 (16.1) | Ref |  | Ref |  |
| Normal, 1 | 20485 (12.6) | 0.69  (0.64 to 0.75) | < 0.001 | 0.30**^*^**  (0.25 to 0.35) | < 0.001 |
| Normal, 2 | 6350 (3.9) | 1.26 (1.18 to 1.34) | < 0.001 | 0.55**^*^**  (0.46 to 0.63) | < 0.001 |
| Normal, 3 | 479 (0.3) | 1.34  (1.07 to 1.61) | < 0.001 | 0.46**^*^**  (0.19 to 0.72) | < 0.001 |
| Overweight, 0 | 18865 (11.6) | 0.49  (0.43 to 0.55) | < 0.001 | 0.32**^*^**  (0.26 to 0.37) | < 0.001 |
| Overweight, 1 | 29517 (18.2) | 1.21  (1.16 to 1.26) | < 0.001 | 0.71**^*^**  (0.66 to 0.75) | < 0.001 |
| Overweight, 2 | 18832 (11.6) | 1.75  (1.69 to 1.80) | < 0.001 | 0.95**^*^**  (0.90 to 1.01) | < 0.001 |
| Overweight, 3 | 1858 (1.1) | 1.92  (1.78 to 2.06) | < 0.001 | 1.02**^*^**  (0.88 to 1.16) | < 0.001 |
| Obesity, 0 | 5324 (3.3) | 0.69  (0.60 to 0.77) | < 0.001 | 0.65**^*^**  (0.57 to 0.74) | < 0.001 |
| Obesity, 1 | 15195 (9.3) | 1.31  (1.25 to 1.37) | < 0.001 | 1.00**^*^**  (0.94 to 1.05) | < 0.001 |
| Obesity, 2 | 15819 (9.7) | 1.78  (1.72 to 1.84) | < 0.001 | 1.18**^*^**  (1.12 to 1.23) | < 0.001 |
| Obesity, 3 | 3345 (2.1) | 1.72  (1.62 to 1.83) | < 0.001 | 0.99**^*^**  (0.88 to 1.09) | < 0.001 |

Regression coefficients (β) were estimated using linear regression.

**^†^**Adjusted for age, sex, smoking, ethnicity, Townsend deprivation quintiles
**^*^**Variance inflation factor (VIF) < 4

BMI, body mass index; CI, confidence interval; MHN, metabolically healthy normal weight; MUN, metabolically unhealthy normal weight; MHOW, metabolically healthy overweight; MUOW, metabolically unhealthy overweight; MHO, metabolically healthy obesity; MUO, metabolically unhealthy obesity.

**Table S3** Relationship between arterial stiffness index, BMI phenotypes, and metabolic score after adjusting for physical activity, alcohol intake frequency and sleep duration.

| **Clinical Variables** | **N (%)** | **Adjusted^†^** | | **Adjusted^¶^** | | **Adjusted^Ø^** | |
| --- | --- | --- | --- | --- | --- | --- | --- |
|  |  | **β (95% CI)** | **p-value** | **β (95% CI)** | **p-value** | **β (95% CI)** | **p-value** |
| **BMI** | | | | | | | |
| Normal | 53835 (33.1) | Ref |  | Ref |  | Ref |  |
| Overweight | 69072 (42.5) | 0.50^*^  (0.46 to 0.54) | < 0.001 | 0.50*  (0.47 to 0.54) | < 0.001 | 0.50*  (0.47 to 0.54) | < 0.001 |
| Obesity | 39683 (24.4) | 0.83^*^  (0.79 to 0.87) | < 0.001 | 0.83*  (0.79 to 0.87) | < 0.001 | 0.84*  (0.79 to 0.88) | < 0.001 |
| **Metabolic Score** | | | | | |  |  |
| 0 | 50710 (31.2) | Ref |  | Ref |  | Ref |  |
| 1 | 65197 (40.1) | 0.47^*^  (0.43 to 0.51) | < 0.001 | 0.47*  (0.43 to 0.51) | < 0.001 | 0.47*  (0.43 to 0.51) | < 0.001 |
| 2 | 41001 (25.2) | 0.80^*^  (0.75 to 0.84) | < 0.001 | 0.80*  (0.75 to 0.84) | < 0.001 | 0.80*  (0.75 to 0.84) | < 0.001 |
| 3 | 5682 (3.5) | 0.70^*^  (0.61 to 0.79) | < 0.001 | 0.70*  (0.61 to 0.79) | < 0.001 | 0.70*  (0.61 to 0.79) | < 0.001 |
| **Metabolic Categories**^¥^ | | | | | | | |
| MHN | 26521 (16.3) | Ref |  | Ref |  | Ref |  |
| MUN | 27314 (16.8) | 0.36^*^  (0.30 to 0.41) | < 0.001 | 0.36*  (0.30 to 0.41) | < 0.001 | 0.36*  (0.30 to 0.41) | < 0.001 |
| MHOW | 18865 (11.6) | 0.34^*^  (0.28 to 0.40) | < 0.001 | 0.34*  (0.28 to 0.40) | < 0.001 | 0.34*  (0.28 to 0.40) | < 0.001 |
| MUOW | 50207 (30.9) | 0.83^*^  (0.79 to 0.88) | < 0.001 | 0.84* (0.79 to 0.89) | < 0.001 | 0.84* (0.79 to 0.89) | < 0.001 |
| MHO | 5324 (3.3) | 0.69^*^  (0.60 to 0.79) | < 0.001 | 0.70*  (0.60 to 0.79) | < 0.001 | 0.70*  (0.60 to 0.79) | < 0.001 |
| MUO | 34359 (21.1) | 1.08^*^  (1.02 to 1.13) | < 0.001 | 1.08*  (1.03 to 1.14) | < 0.001 | 1.08*  (1.03 to 1.14) | < 0.001 |
| **BMI Phenotypes and Metabolic Score** | | | | | | | |
| Normal, 0 | 26521 (16.1) | Ref |  | Ref |  | Ref |  |
| Normal, 1 | 20485 (12.6) | 0.30^*^  (0.25 to 0.36) | < 0.001 | 0.30*  (0.25 to 0.36) | < 0.001 | 0.30*  (0.25 to 0.36) | < 0.001 |
| Normal, 2 | 6350 (3.9) | 0.56^*^  (0.48 to 0.65) | < 0.001 | 0.57*  (0.48 to 0.65) | < 0.001 | 0.56*  (0.48 to 0.65) | < 0.001 |
| Normal, 3 | 479 (0.3) | 0.48^*^  (0.19 to 0.77) | 0.001 | 0.49*  (0.20 to 0.78) | < 0.001 | 0.49*  (0.20 to 0.78) | < 0.001 |
| Overweight, 0 | 18865 (11.6) | 0.34^*^  (0.28 to 0.40) | < 0.001 | 0.34*  (0.29 to 0.40) | < 0.001 | 0.34*  (0.29 to 0.40) | < 0.001 |
| Overweight, 1 | 29517 (18.2) | 0.75^*^  (0.69 to 0.80) | < 0.001 | 0.75*  (0.70 to 0.80) | < 0.001 | 0.75*  (0.70 to 0.80) | < 0.001 |
| Overweight, 2 | 18832 (11.6) | 0.99^*^  (0.93 to 1.05) | < 0.001 | 0.99*  (0.93 to 1.05) | < 0.001 | 0.99*  (0.93 to 1.05) | < 0.001 |
| Overweight, 3 | 1858 (1.1) | 1.03^*^  (0.87 to 1.18) | < 0.001 | 1.04*  (0.88 to 1.19) | < 0.001 | 1.04*  (0.89 to 1.19) | < 0.001 |
| Obesity, 0 | 5324 (3.3) | 0.69^*^  (0.60 to 0.79) | < 0.001 | 0.70*  (0.61 to 0.79) | < 0.001 | 0.70*  (0.60 to 0.79) | < 0.001 |
| Obesity, 1 | 15195 (9.3) | 1.02^*^  (0.96 to 1.08) | < 0.001 | 1.03*  (0.96 to 1.09) | < 0.001 | 1.03*  (0.96 to 1.09) | < 0.001 |
| Obesity, 2 | 15819 (9.7) | 1.19^*^  (1.12 to 1.25) | < 0.001 | 1.20*  (1.13 to 1.26) | < 0.001 | 1.20*  (1.13 to 1.26) | < 0.001 |
| Obesity, 3 | 3345 (2.1) | 0.90^*^  (0.78 to 1.02) | < 0.001 | 0.91*  (0.79 to 1.03) | < 0.001 | 0.91*  (0.79 to 1.03) | < 0.001 |

Regression coefficients (β) were estimated using multivariate-adjusted linear regression.

**^†^**Adjusted for age, sex, smoking, ethnicity, Townsend deprivation quintiles, and physical activity (Summed MET minutes per week for all activity).

**^¶^**Adjusted for age, sex, smoking, ethnicity, Townsend deprivation quintiles, and physical activity (Summed MET minutes per week for all activity), alcohol intake frequency.

**^Ø^**Adjusted for age, sex, smoking, ethnicity, Townsend deprivation quintiles, and physical activity (Summed MET minutes per week for all activity), alcohol intake frequency and sleep duration.

**^*^**Variance inflation factor (VIF) < 4

BMI, body mass index; CI, confidence interval; MHN, metabolically healthy normal weight; MUN, metabolically unhealthy normal weight; MHOW, metabolically healthy overweight; MUOW, metabolically unhealthy overweight; MHO, metabolically healthy obesity; MUO, metabolically unhealthy obesity.

**Table S4** Relationship between arterial stiffness index, BMI phenotypes, and metabolic score after adjusting for metabolic health profile.

| **Clinical Variables** | **N (%)** | **Adjusted** | |
| --- | --- | --- | --- |
|  |  | **β (95% CI)** | **p-value** |
| **BMI** | | | |
| Normal | 53835 (33.1) | Ref |  |
| Overweight | 69072 (42.5) | 0.32*  (0.28 to 0.35) | < 0.001 |
| Obesity | 39683 (24.4) | 0.52*  (0.48 to 0.57) | < 0.001 |
| **Metabolic Score** | | | |
| 0 | 50710 (31.2) | Ref |  |
| 1 | 65197 (40.1) | 0.16*  (0.12 to 0.21) | < 0.001 |
| 2 | 41001 (25.2) | 0.24*  (0.18 to 0.30) | < 0.001 |
| 3 | 5682 (3.5) | 0.03*  (-0.08 to 0.15) | 0.572 |
| **Metabolic Categories**^¥^ | | | |
| MHN | 26521 (16.3) | Ref |  |
| MUN | 27314 (16.8) | 0.11*  (0.05 to 0.17) | < 0.001 |
| MHOW | 18865 (11.6) | 0.28*  (0.22 to 0.35) | < 0.001 |
| MUOW | 50207 (30.9) | 0.43*  (0.37 to 0.49) | < 0.001 |
| MHO | 5324 (3.3) | 0.54*  (0.44 to 0.64) | < 0.001 |
| MUO | 34359 (21.1) | 0.62*  (0.56 to 0.69) | < 0.001 |
| **BMI Phenotypes and Metabolic Score** | | | |
| Normal, 0 | 26521 (16.1) | Ref |  |
| Normal, 1 | 20485 (12.6) | 0.10*  (0.03 to 0.16) | 0.003 |
| Normal, 2 | 6350 (3.9) | 0.17*  (0.07 to 0.26) | < 0.001 |
| Normal, 3 | 479 (0.3) | 0.02*  (-0.28 to 0.32) | 0.888 |
| Overweight, 0 | 18865 (11.6) | 0.28*  (0.22 to 0.35) | < 0.001 |
| Overweight, 1 | 29517 (18.2) | 0.41*  (0.35 to 0.47) | < 0.001 |
| Overweight, 2 | 18832 (11.6) | 0.47*  (0.40 to 0.55) | < 0.001 |
| Overweight, 3 | 1858 (1.1) | 0.42*  (0.25 to 0.59) | < 0.001 |
| Obesity, 0 | 5324 (3.3) | 0.54*  (0.43 to 0.64) | < 0.001 |
| Obesity, 1 | 15195 (9.3) | 0.66*  (0.58 to 0.73) | < 0.001 |
| Obesity, 2 | 15819 (9.7) | 0.65*  (0.58 to 0.73) | < 0.001 |
| Obesity, 3 | 3345 (2.1) | 0.34*  (0.20 to 0.48) | < 0.001 |

Regression coefficients (β) were estimated using linear regression and were adjusted for age, sex, smoking, ethnicity, Townsend deprivation quintiles, systolic blood pressure, HDL, Triglycerides, and HbA1C.

**^*^**Variance inflation factor (VIF) < 4

BMI, body mass index; CI, confidence interval; MHN, metabolically healthy normal weight; MUN, metabolically unhealthy normal weight; MHOW, metabolically healthy overweight; MUOW, metabolically unhealthy overweight; MHO, metabolically healthy obesity; MUO, metabolically unhealthy obesity.

**Table S5** Interaction between BMI phenotypes and metabolic score and its effect on arterial stiffness index.

| **Clinical Variables^†^** | **P-value** |
| --- | --- |
| Overweight | < 0.001 |
| Obesity | < 0.001 |
| Metabolic score 1 | < 0.001 |
| Metabolic score 2 | < 0.001 |
| Metabolic score 3 | < 0.001 |
| Obesity: Metabolic score 2 | 0.009 |
| Obesity: Metabolic score 3 | 0.047 |

^¥^ The linear regression model generated above represents: Arterial stiffness index (Y) = α + β1 x Body-mass index + β2 × metabolic score + β3 × body-mass index*metabolic score

**^†^** Only clinical variables with significant p-value (p < 0.05) were presented in the table above.

**^†^** Obesity: Metabolic score 2 represents interaction between obesity and metabolic score of 2; Obesity: Metabolic score 3 represents interaction between obesity and metabolic score of 3.

**Table S6** Relationship between arterial stiffness index, abdominal obesity and metabolic categories.

| **Clinical Variables** | **N (%)** | **β^†^ (95% CI)** | **p-value** |
| --- | --- | --- | --- |
| **Abdominal Obesity** | | | |
| No | 107619 (66.2) | Ref |  |
| Yes | 54935 (33.8) | 0.66**^*^**  (0.63 to 0.69) | < 0.001 |
| **Metabolic Categories and Abdominal Obesity**^¥^ | | | |
| MHN without abdominal obesity | 26125 (16.1) | Ref |  |
| MHN with abdominal obesity | 394 (0.2) | 0.65**^*^**  (0.36 to 0.93) | < 0.001 |
| MUN without abdominal obesity | 26477 (16.3) | 0.34**^*^**  (0.29 to 0.39) | < 0.001 |
| MUN with abdominal obesity | 834 (0.5) | 1.07**^*^**  (0.87 to 1.27) | < 0.001 |
| MHOW without abdominal obesity | 14938 (9.2) | 0.25**^*^**  (0.19 to 0.31) | < 0.001 |
| MHOW with abdominal obesity | 3925 (2.4) | 0.59**^*^**  (0.50 to 0.69) | < 0.001 |
| MUOW without abdominal obesity | 35232 (21.7) | 0.67**^*^**  (0.62 to 0.72) | < 0.001 |
| MUOW with abdominal obesity | 14966 (9.2) | 1.11**^*^**  (1.05 to 1.16) | < 0.001 |
| MHO without abdominal obesity | 975 (0.6) | 0.42**^*^**  (0.23 to 0.60) | < 0.001 |
| MHO with abdominal obesity | 4344 (2.7) | 0.71**^*^**  (0.62 to 0.81) | < 0.001 |
| MUO without abdominal obesity | 3872 (2.4) | 1.10**^*^**  (1.05 to 1.15) | < 0.001 |
| MUO with abdominal obesity | 30472 (18.7) | 0.89**^*^**  (0.79 to 0.99) | < 0.001 |

Regression coefficients (β) were estimated using linear regression.

**^†^**Adjusted for age, sex, smoking, ethnicity, Townsend deprivation quintiles
**^*^**Variance inflation factor (VIF) < 4

CI, confidence interval; MHN, metabolically healthy normal weight; MUN, metabolically unhealthy normal weight; MHOW, metabolically healthy overweight; MUOW, metabolically unhealthy overweight; MHO, metabolically healthy obesity; MUO, metabolically unhealthy obesity.

**Table S7** Relationship between prevalence of arterial stiffening and BMI phenotypes and metabolic abnormalities.

| **Clinical Variables** | **Prevalence of Arterial Stiffening,**  **N (%)** | **Unadjusted** | | **Adjusted^†^** | |
| --- | --- | --- | --- | --- | --- |
|  |  | **OR (95% CI)** | **p-value** | **OR (95% CI)** | **p-value** |
| **BMI** | | | | | |
| Normal | 16159 (30.0) | Ref |  | Ref |  |
| Overweight | 28279 (40.9) | 1.115  (1.109 to 1.122) | < 0.001 | 1.07  (1.06 to 1.08) | < 0.001 |
| Obesity | 17563 (44.3) | 1.153  (1.146 to 1.160) | < 0.001 | 1.12  (1.11 to 1.13) | < 0.001 |
| **Metabolic Score** | | | | | |
| 0 | 14038 (27.7) | Ref |  | Ref |  |
| 1 | 25524 (39.1) | 1.121  (1.115 to 1.128) | < 0.001 | 1.06  (1.06 to 1.07) | < 0.001 |
| 2 | 19709 (48.1) | 1.226  (1.219 to 1.234) | < 0.001 | 1.11  (1.11 to 1.12) | < 0.001 |
| 3 | 2730 (48.0) | 1.226  (1.210 to 1.242) | < 0.001 | 1.10  (1.09 to 1.12) | < 0.001 |
| **Metabolic Categories**^¥^ | | | | | |
| MHN | 6416 (24.2) | Ref |  | Ref |  |
| MUN | 9743 (35.7) | 1.122  (1.113 to 1.131) | < 0.001 | 1.04  (1.03 to 1.05) | < 0.001 |
| MHOW | 5865 (31.1) | 1.071  (1.062 to 1.081) | < 0.001 | 1.05  (1.04 to 1.05) | < 0.001 |
| MUOW | 22414 (44.6) | 1.227  (1.218 to 1.236) | < 0.001 | 1.12  (1.11 to 1.13) | < 0.001 |
| MHO | 1757 (33.0) | 1.092  (1.077 to 1.108) | < 0.001 | 1.09  (1.07 to 1.10) | < 0.001 |
| MUO | 15806 (46.0) | 1.244  (1.234 to 1.253) | < 0.001 | 1.16  (1.15 to 1.17) | < 0.001 |
| **BMI Phenotypes with Metabolic Score** | | | | | |
| Normal, 0 | 6416 (24.2) | Ref |  | Ref |  |
| Normal, 1 | 6929 (33.8) | 1.101  (1.092 to 1.111) | < 0.001 | 1.04  (1.03 to 1.05) | < 0.001 |
| Normal, 2 | 2619 (41.2) | 1.186  (1.171 to 1.202) | < 0.001 | 1.07  (1.05 to 1.08) | < 0.001 |
| Normal, 3 | 195 (40.8) | 1.180  (1.130 to 1.232) | < 0.001 | 1.03  (0.99 to 1.08) | 0.114 |
| Overweight, 0 | 5865 (31.1) | 1.071  (1.062 to 1.081) | < 0.001 | 1.05  (1.04 to 1.06) | < 0.001 |
| Overweight, 1 | 12180 (41.3) | 1.186  (1.178 to 1.196) | < 0.001 | 1.10  (1.09 to 1.11) | < 0.001 |
| Overweight, 2 | 9267 (49.2) | 1.284  (1.273 to 1.296) | < 0.001 | 1.14  (1.13 to 1.15) | < 0.001 |
| Overweight, 3 | 967 (52.0) | 1.321  (1.292 to 1.351) | < 0.001 | 1.16  (1.13 to 1.18) | < 0.001 |
| Obesity, 0 | 1757 (33.0) | 1.092  (1.077 to 1.108) | < 0.001 | 1.09  (1.07 to 1.10) | < 0.001 |
| Obesity, 1 | 6415 (42.2) | 1.198  (1.186 to 1.209) | < 0.001 | 1.14  (1.13 to 1.15) | < 0.001 |
| Obesity, 2 | 7823 (49.5) | 1.287  (1.275 to 1.300) | < 0.001 | 1.18  (1.17 to 1.19) | < 0.001 |
| Obesity, 3 | 1568 (46.9) | 1.255  (1.233 to 1.276) | < 0.001 | 1.13  (1.11 to 1.15) | < 0.001 |

Odd ratios were estimated using logistic regression.

**^†^**Adjusted for age, sex, smoking, ethnicity, and Townsend deprivation quintiles.

BMI, body mass index; OR, odd ratio; CI, confidence interval; MHN, metabolically healthy normal weight; MUN, metabolically unhealthy normal weight; MHOW, metabolically healthy overweight; MUOW, metabolically unhealthy overweight; MHO, metabolically healthy obesity; MUO, metabolically unhealthy obesity.
